# The sex-biased brain: sexual dimorphism in gene expression in two species of songbirds

**DOI:** 10.1186/1471-2164-12-37

**Published:** 2011-01-14

**Authors:** Sara Naurin, Bengt Hansson, Dennis Hasselquist, Yong-Hwan Kim, Staffan Bensch

**Affiliations:** 1 Department of Biology, Lund University, Ecology Building, S-223 62 Lund, Sweden; 2 Buck institute for age research, 8001 Redwood blvd, Novato, CA 94945, USA

## Abstract

**Background:**

Despite virtually identical DNA sequences between the sexes, sexual dimorphism is a widespread phenomenon in nature. To a large extent the systematic differences between the sexes must therefore arise from processes involving gene regulation. In accordance, sexual dimorphism in gene expression is common and extensive. Genes with sexually dimorphic regulation are known to evolve rapidly, both in DNA sequence and in gene expression profile. Studies of gene expression in related species can shed light on the flexibility, or degree of conservation, of the gene expression profiles underlying sexual dimorphism.

**Results:**

We have studied the extent of sexual dimorphism in gene expression in the brain of two species of songbirds, the zebra finch (*Taeniopygia guttata*) and the common whitethroat (*Sylvia communis*), using large-scale microarray technology. Sexual dimorphism in gene expression was extensive in both species, and predominantly sex-linked: most genes identified were male-biased and Z-linked. Interestingly, approximately 50% of the male-biased Z-linked genes were sex-biased only in one of the study species.

**Conclusion:**

Our results corroborate the results of recent studies in chicken and zebra finch which have been interpreted as caused by a low degree of dosage compensation in female birds (i.e. the heterogametic sex). Moreover, they suggest that zebra finches and common whitethroats dosage compensate partly different sets of genes on the Z chromosome. It is possible that this pattern reflects differences in either the essentiality or the level of sexual antagonism of these genes in the respective species. Such differences might correspond to genes with different rates of evolution related to sexual dimorphism in the avian brain, and might therefore be correlated with differences between the species in sex-specific behaviours.

## Background

Sexual dimorphism, i.e. systematic differences between the sexes within a species, is a well known phenomenon that occurs in most taxa. Some of the more conspicuous examples of sexual dimorphism are the appearance of miniature parasitic males in anglerfish, the tail of the peacock, and the song of the male nightingale (reviewed in [1]). Sexual dimorphism occurs even though the sexes have virtually identical DNA sequences. Hence, sexual dimorphism must in most cases arise due to mechanisms involving gene regulation and gene expression [2,3]. In line with this, a high degree of sex-biased gene expression is a common feature in many different species [3-12].

Sex-biased genes evolve rapidly, both in terms of their DNA sequence and in their gene expression profiles [5,13-19]. Due to their rapid evolution and the fact that they are often involved in reproduction [20-22] or are coding for species specific traits, like for example song and plumage in birds, sex-biased genes are likely to play an important role in sexual selection and speciation [5,23-26]. Accordingly, genes with sex-biased gene expression are often subjected to strong selection [14,15,18,27-29]. Comparisons of gene expression profiles of related species can shed light on the extent of evolution in sex-biased gene expression, and thus give indications of which genes that are involved in the evolution of sex-specific traits during the process of speciation. The sex-biased gene expression in the brain is perhaps of particular interest in this context, because it is linked to behavioural differences between males and females [20-22,30] and thereby to the evolutionary basis of sex-specific behaviours such and how these vary between species.

Genes with sex-biased gene expression are expected to be non-randomly distributed in the genome, with an enrichment of such genes on sex chromosomes (X and Z chromosomes; [5,31,32]). This is due to the sex-bias in the transmission pattern of these chromosomes, where one sex carries two copies and the other sex only one copy. The uneven dose of X and Z chromosomes between the sexes should lead to an accumulation of sexually antagonistic genes on these chromosomes, i.e. genes that are beneficial to one sex but harmful to the other [31-33]. Dominant or partly dominant mutations on X or Z chromosomes are exposed to selection twice as often in the homogametic as in the heterogametic sex. Such a mutation is therefore expected to go to fixation if it is beneficial to homogametic individuals (XX or ZZ) even if it is harmful to the opposite (heterogametic) sex [31,32]. Sex-linked recessive mutations, on the other hand, will always be exposed to selection in the heterogametic sex (XY or ZW). Thus, a recessive antagonistic mutation will readily reach fixation if it is beneficial to heterogametic individuals [31,32]. Once antagonistic alleles have been fixed, selection for down-regulation of such alleles in the sex that carries the cost is expected to occur [5]. This process will induce or increase sex-bias in gene expression and sex chromosomes should thereby be enriched for sex-biased genes [5,26].

Birds are excellent model systems for studies of sexual dimorphism in gene expression due to their extreme sex-dimorphism in morphology and behaviour [1]. At present, large scale genomic resources are only available for two bird species, the chicken (*Gallus gallus*; [34]) and the zebra finch (*Taeniopygia guttata*; http://www.ncbi.nlm.nih.gov/genome/guide). Galliformes and Passeriformes have highly conserved genome structure with few inter-chromosomal rearrangements [35-37], and this opens up the possibility of using the genome structure of the chicken and zebra finch as templates for synteny and gene order for a number of species related to these birds. In the present study, we have used a genome-wide Affymetrix microarray designed for the zebra finch [38] in order to study the extent of sexual dimorphism in gene expression in two passerine birds, the zebra finch and the common whitethroat (*Sylvia communis*). The zebra finch and the common whitethroat are separated by approximately 24-51 million years of evolution [39,40]. The extent to which these two bird species share patterns of sex-biased gene expression in the brain could shed light on the flexibility, or degree of conservation, of the gene expression profiles underlying sexually dimorphic behaviours.

## Methods

### Zebra finch study population and RNA extraction

Total RNA from full telencephalon of 6 female and 6 male zebra finches was used in this study.

Birds were housed at professor Art Arnolds laboratory at University of California, Los Angeles, in indoor flight cages holding 30 same-sexed individuals. Approximately 350 additional birds of both sexes were within visual and acoustic but not physical contact of the study animals. Cages were kept in a light regime of 12 hours of artificial light (07.00-19.00) followed by 12 hours of dark. All birds were hatched at the aviary and sacrificed by decapitation as adults (>90 days of age) by the same person. All birds were healthy at the time of sacrifice (feeding on their own; feathers were not fluffed; keel was not visible through feathers).

Full Telencephalon was removed from the skull intact and flash frozen on dry ice. Samples were stored at -80°C until total RNA was extracted using the protocol for TRI Reagent (Applied Biosystems/Ambion). Whole telencephalon was rapidly lysed (less than 1 minute) using a dounce homogenizer, extracted, precipitated, and re-suspended in DEPC-treated water. All samples were DNase I treated (after extraction from tissue) with Turbo DNase I (Ambion) 37û × 30 and then RNA isolated using a QIAgen RNeasy spin column, eluting with nuclease-free water. Quality of total RNA was determined visually by formaldehyde gel (ribosomal bands showed no evidence of smearing/degradation) and using the Nanodrop ND-1000 spectrophotometer (260/280 ratio >1.9). Extractions and DNase treatment were done randomly to avoid batch effects. Samples were shipped on dry ice to the SCIBLU genomics facility in Lund, Sweden, where hybridizations were performed (see hybridizations below). All samples had good quality total RNA with high and comparable RNA Integrity Numbers (RIN, [41] when tested at SCIBLU (Swegene Center for Integrative Biology at Lund University, genomics, http://www.lth.se/sciblu).

### Common whitethroat study population, field methods and RNA extraction

Total RNA from full brain of 12 female and 12 male common whitethroats (*Sylivia communis*) was used in this study. The common whitethroat is a warbler of the family Sylviidae, a seasonal breeder and a long distance migratory bird. The species breeds in Europe (May-July) and winters in Africa south of the Sahara (October-April). Birds were caught on two locations, in southern Sweden (Skåne: 55°42'16 N, 13°25'52 E) and central Nigeria (Plateau State: 9°2'29 N, 8°58'90 E). Birds from both locations were used in order to increase the sample size as much as possible and in order to avoid producing candidate genes from exclusively breeding or exclusively wintering birds in comparison with the lab-reared zebra finches.

Common whitethroats were caught in the wild, using mist-nets and playback song. Swedish birds (21 birds, 13 males, 8 females) were caught in the end of May 2005 (all birds were caught prior to any egg laying) and Nigerian birds were caught in one session from February to March 2005 and in a second session in January 2006 (22 Nigerian birds were caught, 14 males, 8 females). Birds sacrificed were in good condition (feathers were not ruffled; keel was not visible through the feathers; behavior prior to capture was normal). All birds caught were adults. Samples from 6 male and 6 female birds from each country were included in microarray analyses below.

Birds were sacrificed through decapitation and the entire brain was immediately transferred into a tube containing RNA later™ RNA stabilization Reagent (Qiagen, cat. no: 76106). Samples were kept in the field for 1 to 8 hours (10-25°C), in 4-8°C for one day to three weeks and then in -80°C until extraction. This is within recommendations for RNAlater stabilizing reagent (Qiagen; see the RNAlater Handbook supplied with the buffer).

All Common whitethroat samples were extracted using the RNeasy Lipid Tissue Mini Kit (Qiagen, cat no: 74804). The brains were removed from the RNAlater buffer and the full brain was homogenized in 1 ml QIAzol Lysis Reagent per 100 mg tissue (Qiagen, supplied with kit) using a TissueLyser (Qiagen cat no: 85220). The samples were extracted following the exact instructions in the protocol supplied with the kit (step 9 to 17). Qiagen also offers a RNase-Free DNase set (cat no: 79254) for use as an integrated step in the protocol. All common whitethroat samples were treated with DNase this way.

The brain of each individual was extracted one at a time and quality checked in batches of four. Extractions were randomized to avoid batch effects. Samples were quality checked on a formaldehyde agarose gel (no smearing of ribosomal bands was visible) and 260/280 ratios were checked on an Ultraspec 3000 spectrophotometer (all values were between 2.0 and 2.1).

The correct ethical approvals/permits to sacrifice birds were obtained for both species included in this study.

### The microarray and hybridization

The Lund-zf array is a custom Affymetrix array produced for the Zebra finch; for detailed description see [38]. It contains 23136 ESTs corresponding to about 15800 non-redundant genes. Each EST is represented by 11 (25 bases long) probes (except for 148 ESTs that are represented by 8, 9 or 10 probes) and in total there are 254430 probes on the array. The array contains no Affymetrix Mis-Match (MM) probes [38].

High-quality total-RNA samples representing each individual (24 common whitethroat samples and 12 zebra finch samples) were delivered to an Affymetrix service provider, the Swegene Center for Integrative Biology at Lund University (SCIBLU genomics, http://www.lth.se/sciblu), where they were hybridized according to standard Affymetrix protocols for RNA. Before hybridization they were once again quality-checked at SCIBLU using a Nanodrop spectrophotometer and an Agilent 2100 Bioanalyzer. All samples were of high quality with high and comparable RNA Integrity Numbers (RIN, [41] when quality was checked at the SCIBLU genomics facility in Lund. 5 μg total RNA from each sample was used in the regular protocols for GeneChip Arrays and hybridized onto the Lund-zf Affymetrix array overnight in the GeneChip^® ^Hybridisation oven 6400 using standard procedures. The arrays were washed and then stained in a GeneChip^® ^Fluidics Station 450. These procedures were randomized when possible to avoid batch effects. Scanning was carried out with the GeneChip^® ^Scanner 3000 and image analysis was performed using GeneChip^® ^Operating Software.

Files and details on this experiment can be found at ArrayExpress (http://www.ebi.ac.uk accession nr E-MEXP-2914) Since the Lund-zf array contains no Mis-Match (MM) probes, the CEL-files carry only information about the PM probes corresponding to the ESTs on the array. CEL-files were imported into GeneSpring GX 7.3.1 and RMA normalized. RMA normalization requires no MM probe signals. Signal intensities for all ESTs on the array were then filtered (see below) and Quality control was performed with Expression Console™ 1.0.2467.39138 (Affymetrix) on RMA normalized data. For 34 samples, Affymetrix amplification and hybridization controls showed normal patterns and internal controls showed normal 3'/5' ratios. Correlation plots of biological replicates showed high correlations for both data sets. Two samples showed a somewhat deviating profile, two common whitethroats, one male and one female. In the case of the male, the sample had degraded somewhat between QC controls at Lund University and at SCIBLU. We found no explanation as to why the female sample was deviating but assumed that something in the sample was interfering with hybridization. The two problematic samples were excluded from all downstream analyses.

### Filtering of microarray data and hybridization efficiency

Signal data for all arrays were filtered to remove the ESTs with large standard deviation. This was done to remove any potential noise in the data and all ESTs with standard deviations larger than 30% of the median value for the signal was removed from the analyses. In this filtration, 2055 ESTs were removed from the analysis of zebra finch arrays and 577 ESTs were removed from the analyses of common whitethroat arrays. Furthermore, all common whitethroat arrays were filtered based on the data produced in a Comparative Genome Hybridization, CGH [38]. This was done in order to avoid analysing probes with a high degree of sequence divergence, and 9827 probes that had non-significant signals in the common whitethroat CGH analysis were removed from downstream analyses. These represent all probes in the CGH analyses that did not hybridize significantly when DNA of the common whitethroat was hybridized to the array, but did hybridize significantly when zebra finch DNA was hybridized. The 9827 probes are likely to represent parts of genes on the array that does not function for the common whitethroat due to sequence divergence between the species. The fact that only 9827 out of the ca. 250000 probes (i.e. <4% of the total number of probes) were lost in the CGH analyses means that the cross-species hybridisation in this study should not lead to unreliable results for the whitethroat. All ESTs were flagged according to how many probes they had lost after the 9827 probes were removed. This provided us with a number between 11 (= retained all 11 probes) to 0 (= had lost all 11 probes). Only ESTs retaining at least 8 out of the 11 probes were analysed, the rest (554 ESTs) were removed from all downstream analyses of the common whitethroat arrays. The choice of this cut off was motivated by the fact that Affymetrix normally allows analyses on probe-sets that retain significant signals on at least 8 probes http://www.affymetrix.com/index.affx. 4968 ESTs lost one probe in the filtering above but were still analysed, 1662 lost 2 probes and 595 lost 3 probes. After filtering 21081 ESTs remained to be analysed on the zebra finch arrays and 22005 on the common whitethroat arrays.

The fact that only 554 ESTs were removed in the filtering process above confirms that the common whitethroat samples performed well on the chip [38]. The efficiency of our filtering is further confirmed when hybridization-performance of whitethroat RNA is studied in detail. Out of the 268 ESTs that were sex-biased only in the zebra finch in this study (see below) no more than 14 had been filtered away. When these 14 ESTs were excluded from analyses the remaining 254 ESTs had not lost significantly more probes than the ESTs biased in both species (t-test: p = 0.379). Moreover, even if mean raw signal of the common whitethroat chips (308) was lower than the mean of zebra finch chips (533) prior to filtering mean hybridization signal in the common whitethroat was not significantly different between the different categories of genes identified in this study (genes that were sex-biased only in the finch, genes sex-biased in both species or genes sex-biased only in the whitethroat). In conclusion, even if the common whitethroat has a disadvantage on the array due to sequence divergence, this problem is minor and can be effectively controlled via filtering.

### Significance Analysis of Microarrays

Significance Analysis of Microarray (SAM) is a statistical approach to find genes with significant differences in expression in sets of microarray experiments [42]. Input data is gene expression measurements and response variables for each experiment. SAM computes a statistic d_*i *_for each gene *i*, measuring the strength of the relationship between gene expression and the response variable. It uses permutations to assess which genes are significant. Filtered data was imported into Microsoft Office Excel 2003 and analysed using the Significance Analysis of Microarrays 3.02 plugin. Two class (unpaired) tests were run with False Discovery Rates (FDR) set as close to 3% as possible for all analyses. SAM was set to 500 permutations.

All analyses were done on log 2 signals with the response variable being sex, and separately for the two species. Males were entered as control group and females as treatment group (results are not affected if females are used as control group). No Fold Change (FC) criterion was specified in SAM. This was due to the expected estimates of sexual dimorphism in the brain. For many studies of sexual dimorphism in gene expression, no genes with a FC lower than a prior set level has been listed as significant. This level is often quite high (2-fold; e.g., [19,43]). However, many of these studies have been conducted on gonads, where one would expect the estimates of sex-biased gene expression to be at their highest. In the brain, most gene expression differences between the sexes are not expected to be that high [44]. We have accepted all genes identified by the permutation test in SAM as significant. In order to facilitate comparisons with other studies of gene expression in birds the Fold Changes given here were calculated separately on unlogged data for the significant genes, as mean male-expression over mean female-expression. The genes counted as sex-biased in this study have FCs ranging from 0.5 to 1.2 in the zebra finch and 0.3 to 1.7 in the common whitethroat.

### EST annotation

Estimations of redundancies amongst the ESTs on the array have been made using the annotations produced for each sequence in the ESTIMA: songbird build 2 assembly [45]. The chicken TC-id listed in ESTIMA was compared for all ESTs. ESTs with identical TC-id were listed as the same gene. We then confirmed non-redundancies by BLASTing all our ESTs against the 3.2.4 build of the zebra finch genome. ESTs with the same chicken-TC-id in ESTIMA also had hits against the same chromosome and position in the BLAST. The ESTs with no annotation in the ESTIMA assembly are simply listed as non-redundant genes here. To generate correct information regarding chromosomal location, we used data from the BLAST against the 3.2.4 build of the zebra finch genome mentioned above http://www.ncbi.nlm.nih.gov/genome/guide/finch/ and genes with non significant hits (i.e. with E-values > 1*10^-20^) in this BLAST has been labelled no annotation. This annotation gave 1075 ESTs with significant Z hits on the array (4.6%), 17954 ESTs with significant autosomal hits (77%), and 4107 ESTs with no significant annotation.

## Results

### Zebra finch

**Figure 1:**
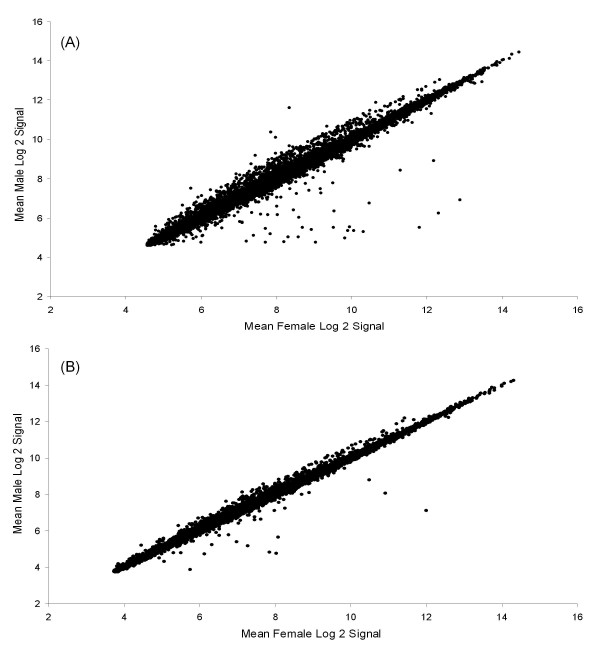
**Gene expression signals in males versus females**. Female gene expression signal versus male signal for all ESTs on the Lund-zf array in (**a**): the zebra finch and (**b**): the common whitethroat.

The male and female expression levels of the 21081 ESTs that remained after filtering in the zebra finch are shown in Figure 1a. The SAM analyses showed that 509 of these 21081 ESTs (i.e. 2.4%) were significantly differentially expressed between the sexes at a false discovery rate of 3.9% (Table 1; delta parameter = 1.28), while the expected number of false discoveries in this data should be less than 20 ESTs. After annotations, the significant ESTs were found to correspond to 417 non-redundant genes (see Additional file 1 for list of genes). Of the 417 identified sex-biased genes, 92% were male-biased in expression (Table 1) and male-biased genes had a mean FC (male over female expression ratio) of 1.08 while female-biased genes had a mean FC of 0.77. Out of the significantly sex-biased genes, 64 were represented by more than one significantly sex-biased EST on the Lund-zf array. Among these 64 genes, 15 were represented by both female-biased and male-biased ESTs (i.e. 'ambiguous' genes); and 14 out of the 15 ambiguous genes were in turn Z-linked. Female-biased ESTs in such genes had lower mean identity to the Z-sequence in the zebra finch draft (90%) then the male-biased parts (98%), indicating that some female-biased ESTs might in fact be sequences from W homologues. When these ESTs are instead blasted against the chicken genome http://www.ncbi.nlm.nih.gov/projects/genome/guide/chicken/, three have significant hits on both Z and W.

**Table 1:** Numbers of sex-biased ESTs and genes identified

	ESTs	non-redundant genes	% of all sex-biased ESTs	% of all sex-biased genes	mean FC non-redundant genes
Zebra Finch					
male-biased genes	460	383	90	95	1.08
female-biased genes	49	19	9.6	4.7	0.77
Common Whitethroat					
male-biased genes	318	271	92	94	1.32
female-biased genes	27	16	7.8	5.6	0.66

A total of 509 ESTs corresponding to 417 genes were identified in the zebra finch, and 345 ESTs corresponding to 299 genes in the common whitethroat.

**Figure 2:**
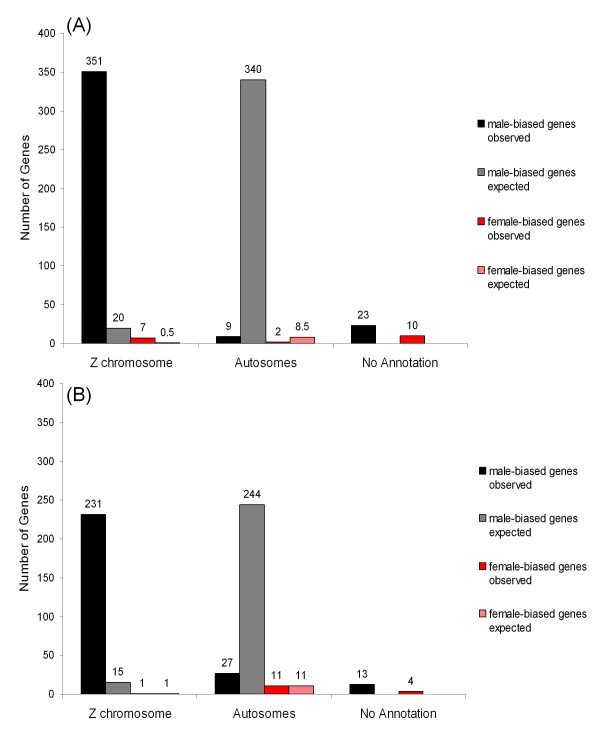
**Histogram showing the observed and expected genomic distribution of sex-biased genes**. The number of sex-biased genes for each chromosomal category in (**a) **zebra finch and (**b**) common whitethroat. Annotations achieved by BLASTs of the EST sequences on the array against the zebra finch sequence assembly (build 3.2.4). Significant hits have a hit of E = 10^-20 ^or lower, and "no annotation" indicates that the EST sequence has either a significant hit against TguUnknown or no significant hit against any of the zebra finch chromosomes. Numbers above bars represent number of genes in each category.

The majority of the 402 non-redundant *and *unambiguous sex-biased genes were Z-linked (351 male-biased and 7 female-biased; Figure 2a), and both male-biased and female-biased genes showed a non-random distribution across chromosomes, with an overrepresentation on the Z chromosome (Table 2).

**Table 2:** Fisher exact test of genomic distributions

	zebra finch observed	common whitethroat observed	Expected if distribution was random
**female-biased genes**			
Z-linked	0.777 (7)	0.056 (1)	0.056
Autosomal	0.222 (2)	0.944 (11)	0.944
p value Fisher exact test	0.0055	NS	
**male-biased genes**			
Z-linked	0.975 (351)	0.895 (231)	0.056
Autosomal	0.0025 (9)	0.105 (27)	0.944
p value Fisher exact test	1.30E-165	3.00E-94	

Observed and expected genomic distribution of all annotated sex-biased genes that show non-ambiguous regulation. Number of genes in parentheses.

### Common whitethroat

The male and female expression levels of the 22005 ESTs that remained after filtering in the common whitethroat are shown in Figure 1b. SAM analyses were performed on all these ESTs and were first run for all Swedish birds (5 males and 6 females) versus all Nigerian birds (6 males and 5 females). A few genes (52) were identified as differentially expressed between the two seasons/populations at false discovery rate 3.8 (delta = 0.61; results not shown) but none of them were identified in the comparison between the sexes, and so the sexual dimorphism identified here is not driven by birds caught in a particular season. In total, 345 of the 22005 ESTs (1.6%) were found to be differentially expressed between the sexes at false discovery rate 3.5 (delta = 0.85) and the expected number of false discoveries in this data should therefore be less than 13 ESTs. After annotation, the significant ESTs were found to correspond to 299 non-redundant genes (see Additional file 2 for list of genes). Of the 299 identified genes, 91% were male-biased in expression (Table 1) and male-biased genes had a mean FC of 1.32 while female-biased genes had a mean FC of 0.66.

Out of the 299 non-redundant sex-biased genes, 36 were found to be represented by more than one significant sex-biased EST on the Lund-zf array; and of these 36, 12 were in turn 'ambiguous', i.e. represented by both female-biased and male-biased ESTs.

Also in the common whitethroat, the majority of the 299 non-redundant and unambiguous sex-biased genes were Z-linked (231 male-biased and 1 female-biased; Figure 2b) and the male-biased genes were significantly non-randomly distributed across chromosomes (Table 2).

### Comparison of sex-biased gene expression in zebra finch and common whitethroat

**Figure 3:**
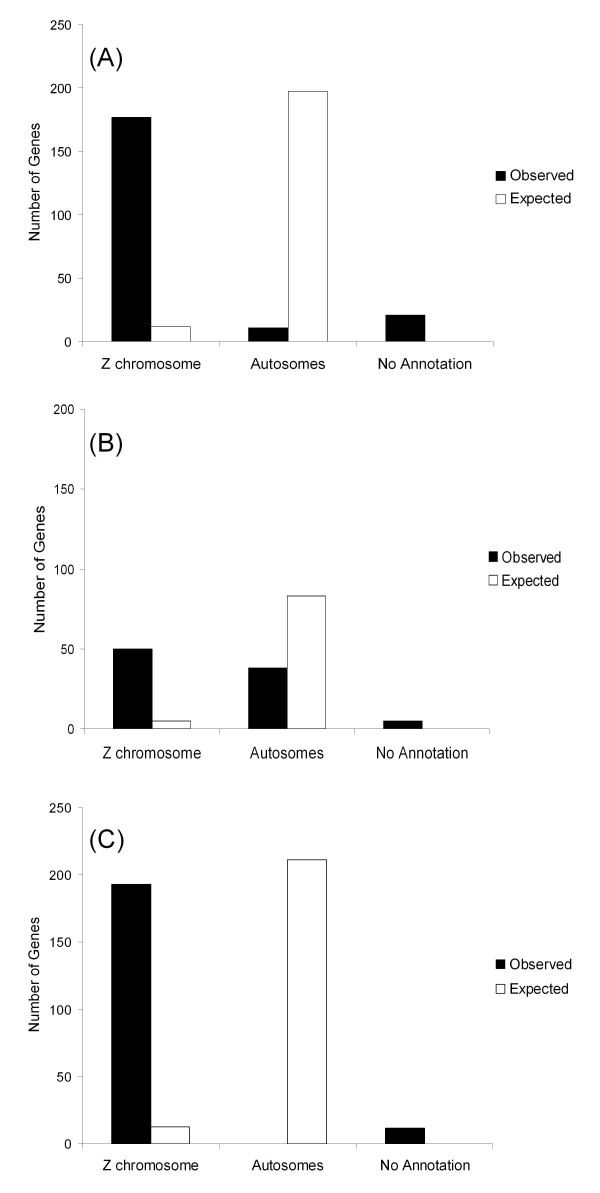
**Genomic distributions of species specific sex-biased genes and of genes sex-biased in both species**. Chromosome annotation of (**a**) the 212 genes which were sex-biased only in the zebra finch, (**b**) the 93 genes that were sex-biased only in the common whitethroat and (**c**) the 205 genes that were sex-biased in both species.

In total, 205 non-redundant genes were identified as differentially expressed between the sexes in both the zebra finch and the common whitethroat (Figure 3; see Additional file 3 for list of genes). These 205 genes were biased for the same sex in both species and 12 of them showed ambiguous regulation (i.e. were represented by both female-biased and male-biased ESTs in both species). In total, 212 non-redundant genes were found to be sex-biased only in the zebra finch (Figure 3a) and 93 were sex-biased only in the common whitethroat (Figure 3b). Only 4 of the 205 genes biased in both species where female-biased and 180 of them (88%) were both male-biased and Z-linked (Figure 3c). The proportion of male-biased and Z-linked genes was lower among the genes only biased in the zebra finch (80%, 171 genes) and in the genes only biased in the common whitethroat (45%, 43 genes). Moreover, there were no autosomal genes that were sex-biased in both species (Figure 3c).

## Discussion

We found extensive sexual dimorphism in gene expression in the brain of both the zebra finch and the common whitethroat. In light of the many differences between the species in terms of evolutionary time since divergence, sexual behaviour and morphology, and in sampling regimes, it is remarkable that almost 50% (205 of 417) of the genes identified as sex-biased in the zebra finch were also identified as sex-biased in the common whitethroat. These 205 genes did not only show sex-bias in both species, none of them showed a reversal in sex-bias; i.e., if they were female-biased in one species they were also female-biased in the other. Differences in gene expression between species and populations can be extensive [5-10,46]. Moreover, sex-biased genes are known to evolve rapidly and to reverse their sex-bias between different species [5]. It is therefore interesting, that the two passerine species studied here show such high similarity in the pattern of sex-biased gene expression.

Nevertheless, although there were substantial the similarities in the results of the two study species, no less than 212 genes were sex-biased only in the zebra finch. These genes could represent hot spots for divergent selection and species-specific evolution of gene regulation on the Z chromosome. They did not have a lower degree of hybridization success in the common whitethroat than genes with significant sex-bias in that species, indicating that they to a large extent represent 'true' differences between the species (as opposed to having been missed in the whitethroat due to sequence dissimilarities). Moreover, 93 genes were identified as sex-biased only in the common whitethroat. The whitethroat samples contained the entire brain while only the telencephalon was hybridized from the zebra finches. This means that 'unique' common whitethroat genes will belong to two categories: (i) telencephalon species-specific genes and (ii) genes implemented in the regulation of sex dimorphism in other parts of the brain.

Male-biased genes dominated the data sets in this study, and this is similar to previous studies in birds [4,12,33]. The Z chromosome was enriched with male-biased genes in both the zebra finch (351 genes) and the common whitethroat (231 genes), and in the zebra finch female-biased genes were also overrepresented on the Z chromosome (7 genes). This is in line with theory predicting pronounced sexual antagonism and thereby an accumulation of genes with sex-biased expression on the Z chromosome (which in the chicken, *Gallus gallus *has a total of 840 genes and in the zebra finch has 717 identified genes so far) [31,32]. However, as previous studies of the zebra finch and the chicken have suggested, birds seem to exhibit incomplete dosage compensation [4,12]. This has important implications for our results, both in terms of the number of male-biased genes and in terms of the similarities between the species.

### Dosage compensation and sexual antagonism on the Z chromosome

A low degree of dosage compensation will lead to a generally higher expression of Z-linked genes in males (the homogametic sex) compared to females (the heterogametic sex), simply due to the double dose of Z in males. It could therefore be possible that the double dose of Z in avian males explains the large number of male-biased Z-linked genes in our data sets. A lack of dosage compensation would make it difficult to separate between (i) genes that are essential to male-specific morphology and behavior and thus being up-regulated in males, and (ii) genes that have male-biased expression due to Z-linkage and a double dose in males.

There was a high proportion of male-biased Z-linked genes among the sex-biased genes, and this was true for both the genes that were biased in both species (88% male-biased and Z-linked), and the genes that were biased only in one of the species (80% in the zebra finch; 45% in the common whitethroat). This suggests that if the high degree of male-bias on the avian Z chromosome is indeed centred around the extent of dosage compensation, then there are species-specific differences in which genes and parts of the Z chromosome that are dosage compensated. This is highly interesting as the extent of compensation could be associated with the extent of sexual dimorphism and species differentiation. Finches and warblers separated 24-51 million years ago [39,40,47] and may have taken different routes during the evolution of sex chromosomes and dosage compensation of essential genes. It is possible, therefore, that some genes are compensated to a higher extent in one species than the other and that this pattern represents the essentiality of the genes in the respective species. Hence, it can be suggested that the differences between the species, whether produced by actual sex-dimorphic regulation or by differences in the level of dosage compensation, reflect the occurrence of species-specific hot spots for the evolution of sex dimorphism in the avian brain, including hot-spots in the evolution of sex-dimorphic behaviors.

If a large proportion of the male-biased genes identified in this study are poorly compensated in females rather than specifically up-regulated by males; that leads to questions regarding detrimental effects of lower Z-linked gene expression in females. Aneuploidy, i.e. having a lower or higher copy number of a part of the genome, is normally lethal due to effects of gene dose on crucial networks [48-52]. In line with this, dosage compensation is wide-spread in other taxa, including mammals [53-55]. It is likely therefore that the most essential genes should show a higher degree of dosage compensation in female birds. There is some evidence that this is the case in chicken [12] but more comprehensive studies are needed also in other bird species. It seems likely that low expression at hundreds of Z-linked loci would have detrimental effect on the female phenotype. If that is the case, then sexual dimorphism in birds is perhaps not selected only via regulation of genes with sex-specific tasks but also via selection for different levels of dosage compensation of Z-linked genes in females. It is important to notice, however, that the mean fold changes for male-biased genes in this study is not very high (1.08 in the zebra finch and 1.32 in the common whitethroat), hence, if a lack dosage compensation has caused sex-biased gene expression on a high number of genes on the Z chromosome in these species then some compensation seems to have occurred.

Another potential explanation for a large amount of sex-biased gene expression on the Z chromosome relates to sexual antagonisms. If a gene is antagonistic, i.e. its expression is favorable for one sex and harmful for the other, then the sex for which it is harmful would be expected to down-regulate the expression of that gene [5,26,31,32]. This down-regulation will induce or increase sex-bias. Theory predicts that antagonistic genes should aggregate on the Z (or X) chromosomes. This is due to the uneven dose of sex chromosomes between the sexes, which will lead to selection and fixation of dominant mutations in males and recessive mutations in females [31,32]. How much such antagonism contributes to sex-bias in the avian brain is difficult to say at present. However, a recent study identified quite a large number of sexually antagonistic genes on the Z-chromosome in chicken [56]. Moreover, male-biased genes belong to different functional categories than unbiased genes [57] and are expressed at different levels [57], indicating systematic differences between these two types of Z-linked avian genes.

## Conclusions

We found a high degree of sexually dimorphic gene expression in the brain of two passerine birds. Given that the brain is not expected to be nearly as sexually dimorphic in gene expression as specialized tissues, like testes and ovaries, the identification of several hundreds of genes with significantly different expression between the sexes is highly interesting and implies that the avian brain is truly affected by the sex of brain cells. These and other recent results on gene expression in birds [4,12,56,58] lead to questions concerning to what extent the pronounced sexual dimorphism in morphology and behavior in birds can be attributed to the polarization of sexually dependent gene expression in the avian brain. Moreover, our results suggest that even though a very high degree of male-biased gene expression on the Z chromosome is the common pattern in birds, species differ quite substantially in regard to which genes that are male-biased. The difference between the species in which genes that are dosage compensated does thereby seem to reflect differences between the species in levels of antagonism and essentiality of Z-linked genes.

## Authors' contributions

SN had the main responsibility for the whole study, including constructing the microarray, designing the study, collecting samples from common whitethroats, RNA lab work, analyzing and interpreting data, as well as writing the manuscript. BH, DH, SB participated in the design of the microarray, in the planning of the study, in the interpreting and discussions regarding the results, as well as in the writing of the manuscript. YHK prepared and provided the zebra finch samples, participated in the discussion of results and in the writing of the manuscript. All authors read and approved the final manuscript.

## Supplementary Material

Additional file 1 **List of genes sex-biased in zebra finch**. Information regarding all ESTs and genes identified as sex-dimorphic in the zebra finch. Click here for file

Additional file 2 **List of genes sex-biased in common white throat**. Information regarding all ESTs and genes identified as sex-dimorphic in the common whitethroat. Click here for file

Additional file 3 **List of genes sex-biased in both species**. Information regarding all ESTs and genes identified as sex-dimorphic in both species. Click here for file
